# Effects of prostacyclin on the early inflammatory response in patients with traumatic brain injury-a randomised clinical study

**DOI:** 10.1186/2193-1801-3-98

**Published:** 2014-02-18

**Authors:** Marie Rodling Wahlström, Magnus Olivecrona, Clas Ahlm, Anders Bengtsson, Lars-Owe D Koskinen, Silvana Naredi, Magnus Hultin

**Affiliations:** Department of Surgical and Perioperative Sciences, Anaesthesiology and Intensive Care, Umeå University, S-901 87 Umeå, Sweden; Department of Pharmacology and Clinical Neurosciences, Neurosurgery, Umeå University, S-901 87 Umeå, Sweden; Department of Clinical Microbiology, Infectious Diseases, Umeå University, S-901 87 Umeå, Sweden; Institute of Clinical Sciences, Department of Anesthesiology and Intensive Care, The Sahlgrenska Academy, University of Gothenburg, S-405 30 Gothenburg, Sweden

**Keywords:** Traumatic brain injury, Epoprostenol, Systemic inflammatory response, Cytokines, Asymmetric dimethylarginine

## Abstract

**Objective and design:**

A prospective, randomised, double-blinded, clinical trial was performed at a level 1 trauma centre to determine if a prostacyclin analogue, epoprostenol (Flolan®), could attenuate systemic inflammatory response in patients with severe traumatic brain injury (TBI).

**Subjects:**

46 patients with severe TBI, randomised to epoprostenol (n = 23) or placebo (n = 23).

**Treatment:**

Epoprostenol, 0.5 ng · kg^-1^ · min^-1^, or placebo (saline) was given intravenously for 72 hours and then tapered off over the next 24 hours.

**Methods:**

Interleukin-6 (IL-6), interleukin-8 (IL-8), soluble intracellular adhesion molecule-1 (sICAM-1), C-reactive protein (CRP), and asymmetric dimethylarginine (ADMA) levels were measured over five days. Measurements were made at 24 h intervals ≤24 h after TBI to 97–120 h after TBI.

**Results:**

A significantly lower CRP level was detected in the epoprostenol group compared to the placebo group within 73–96 h (*p* = 0.04) and within 97–120 h (*p* = 0.008) after trauma. IL-6 within 73–96 h after TBI was significantly lower in the epoprostenol group compared to the placebo group (*p* = 0.04). ADMA was significantly increased within 49–72 h and remained elevated, but there was no effect of epoprostenol on ADMA levels. No significant differences between the epoprostenol and placebo groups were detected for IL-8 or sICAM-1.

**Conclusions:**

Administration of the prostacyclin analogue epoprostenol significantly decreased CRP and, to some extent, IL-6 levels in patients with severe TBI compared to placebo. These findings indicate an interesting option for treatment of TBI and warrants future larger studies.

**Trial registration:**

ClinicalTrials.gov Identifier, NCT01363583

## Introduction

Following traumatic injuries with or without severe traumatic brain injury (TBI), an inflammatory response is induced both in the brain and in the systemic circulation (Holmin et al. 1998; Lu et al. 2009). Severe injury is associated with a systemic host-defence reaction – known as systemic inflammatory response syndrome (SIRS) – that can progress into multiple organ failure (Lenz et al. 2007). Experimental studies and clinical studies in trauma patients have shown that increased production of inflammatory mediators is associated with SIRS and subsequent organ failure (Lenz et al. 2007; Stahel et al. 2007), and several different inflammatory mediators have been used to measure the inflammatory response (Lu et al. 2009; Lenz et al. 2007).

Modulating the systemic pro-inflammatory response and reducing cytokine overproduction might be an effective therapeutic strategy for preventing organ failure due to SIRS after traumatic injuries, including severe TBI (Neunaber et al. 2011). Whether reducing the inflammatory response after trauma is an entirely beneficial prospect, however, is an unanswered question (Lenz et al. 2007). The endogenous substance prostacyclin, which is released from vascular endothelial cells, has the potential to alter the systemic inflammatory response. In experimental studies, prostacyclin has been shown to inhibit leucocyte activation and adhesion to the endothelium as well as to reduce already established leucocyte adhesion. Prostacyclin has also been suggested to scavenge oxygen free radicals and to reduce the synthesis of mediators of the SIRS cascade (Moncada et al. 1976; Jones and Hurley 1984; Scheeren and Radermacher 1997). Reduced production and/or effects of endogenous prostacyclin due to an imbalance in the prostaglandin-thromboxane pathways have been found experimentally to be associated with TBI (Orfeo et al. 1994).

In this study, interleukin-6 (IL-6), interleukin-8 (IL-8), soluble intracellular adhesion molecule-1 (sICAM-1), and C-reactive protein (CRP) were used as markers for the systemic inflammatory response. IL-6 is one of the most studied pro-inflammatory cytokines after trauma (Jawa et al. 2011). IL-8 is produced by a large variety of cells, including macrophages and monocytes, and functions as a chemotactic cytokine by drawing neutrophils to the site of tissue injury or inflammation (Charo and Ransohoff 2006). The adhesion molecule sICAM-1 forms a connection between leucocytes and the endothelium and is necessary for adequate transmigration of leucocytes through the endothelium. This molecule, therefore, reflects the degree of endothelial cell activation (Lawson and Wolf 2009). CRP is a well known and widely used non-specific marker for inflammation (Du Clos 2000). In addition, asymmetric dimethylarginine (ADMA), an endogenous inhibitor of nitric oxide, was used as a marker for endothelial dysfunction (Böger 2003).

The aim of this study was to evaluate the effect of epoprostenol versus placebo on systemic inflammatory response and endothelial dysfunction during the initial five days after severe TBI. The hypothesis was that epoprostenol could suppress the early systemic pro-inflammatory response after trauma.

## Patients and methods

### Study design

This prospective, double-blinded, randomised, clinical study was conducted with a primary aim of evaluating the cerebral microcirculatory effect of a prostacyclin analogue (epoprostenol, Flolan®, Glaxo SmithKline) compared to placebo (saline) in the injured brain. Results on cerebral microcirculation and clinical outcome have been published previously (Olivecrona et al. 2009). The part of the study reported here evaluates the effects of epoprostenol on the systemic inflammatory response and endothelial function by measuring the levels of CRP, IL-6, IL-8, sICAM-1, and ADMA. The study has been registered as a drug study; ClinicalTrials.gov Identifier: NCT01363583 ( http://clinicaltrials.gov/ct2/show/NCT01363583).

Randomization was performed by means of the random number method. All staff members and investigators were blinded as to treatment group throughout the study period. Patients with severe TBI admitted to the Intensive Care Unit (ICU) at Umeå University Hospital, Sweden between January 1, 2002, and December 31, 2005, were eligible to be recruited into the study if they fulfilled the inclusion criteria below.

The levels of the inflammatory markers CRP, IL-6, IL-8, sICAM-1, and ADMA (as a marker for endothelial dysfunction) were measured daily between 9 a.m. and 12 a.m.

### Ethics

The Local Research Ethics Committee of Umeå University, Sweden, approved the study protocol and, because the study was classified as a drug study, it was also approved by the Medical Products Agency of Sweden. Written informed consent was obtained upon arrival from next of kin. Delayed informed consent from the patient was obtained at the three-month follow-up if possible.

### Inclusion and exclusion criteria

The inclusion criteria were severe TBI defined as Glasgow Coma Scale (GCS) score ≤8 at the time of sedation and intubation, age between 15 years and 70 years, arrival at Umeå University Hospital within 24 hours from the accident, and cerebral perfusion pressure (CPP) ≥10 mmHg at first measurement.

Exclusion criteria were pregnant or lactating women, known bleeding diathesis, known allergy to epoprostenol, penetrating head trauma, or being discharged alive from the ICU within 72 hours.

### Treatment

An intracranial pressure targeted treatment protocol, based on the Lund concept, was used. This treatment approach for severe TBI has been thoroughly described in previous publications (Grände 2006; Naredi et al. 2001). Normovolemia and normotension was aggressively maintained and no hypotensive events were recorded during administration of study drugs, neither in the control group nor in the intervention group.

### Scoring

The scoring systems used to describe the severity of injury included the GCS, the Injury Severity Score (ISS), the Acute Physiologic and Chronic Health Evaluation II (APACHE II), and the Sequential Organ Failure Assessment (SOFA) (Teasdale and Jennett 1974; Baker et al. 1974; Knaus et al. 1985; Vincent et al. 1996). GCS was collected before sedation and intubation. The SOFA score evaluates the degree of organ failure in six different systems (respiratory, haematological, hepatic, cardiovascular, renal, and central nervous system) from zero (no organ failure) to four (severe organ failure). The worst parameter per day was registered without consideration to duration. Severe organ failure was defined as SOFA ≥3 (Knaus et al. 1985).

### Drug

Prostacyclin (epoprostenol, Flolan®, Glaxo SmithKline) or placebo (saline) was administered intravenously at rate of 0.5 ng · kg^-1^ · min^-1^. The hospital pharmacy prepared the drugs according to the randomization number and delivered them in identical boxes labelled with test drug and randomization number. The epoprostenol or placebo infusion was started as soon as possible after arrival at the ICU and was continued for 72 hours and then tapered off during the next 24 hours. The endogenous production of prostacyclin has been estimated to be in the range of 60 pg · kg^-1^ · min^-1^ to 0.1 ng · kg^-1^ · min^-1^ (Jones and Hurley 1984; Scheeren and Radermacher 1997). The epoprostenol dose used in this study, 0.5 ng · kg^-1^ · min^-1^, was based on two previous clinical studies, and this dose was not associated with any adverse events as thrombocytopenia, hypotension or increased pulmonary shunt (Naredi et al. 2001; Grände et al. 2000; Chin et al. 2009).

### Data

An ICU monitoring system (Marquette Solar, General Electric Medical Systems) and an ICU medical records computerised system (PICIS Inc., Wakefield, MA, USA) collected all physiological data continuously.

The exact time of the accident was recorded and both the inflammatory markers (CRP, IL-6, IL-8, and sICAM-1) and the marker for endothelial dysfunction (ADMA) were linked to the time of the TBI and the results are presented in 24 h intervals after TBI (≤24 h, 25–48 h, 49–72 h, 73–96 h, and 97–120 h).

Hospital days (0–5) were used for SOFA scoring. The definition of hospital days is based on the fact that all sampling and summation of data at our ICU, including SOFA score, is made in 24 hours intervals starting at 7 a.m. every day. Day zero starts from the hour the patient arrives at the ICU and lasts until 7 a.m. the following morning. Days 1–5 start at 7 a.m. and last for 24 hours.

SOFA was scored daily for respiration, circulation, coagulation, liver, and kidney failure. The central nervous system was not scored because the patients were continuously sedated.

### Cytokines, ADMA, and laboratory parameters

Blood samples for cytokines were worked up immediately after being drawn from the patients. Serum was separated by centrifugation at 3000 rpm at room temperature and samples were frozen within 30 minutes in separate test tubes and stored at -80°C until the assays for cytokines were performed. The levels of serum IL-6, IL-8 (Pierce Biotechnology Inc., Rockford, IL, USA), and sICAM-1 (Biosource International Inc., Camarillo, CA, USA) were determined by double anti-body enzyme linked immunosorbent assays (ELISA) according to the manufacturer’s procedure. All measurements were performed in duplicate. Reference levels, according to the manufacturer, were 43 pg/mL for IL-6, 8.6 pg/mL for IL-8, and 230 ng/mL for sICAM-1.

To analyse ADMA, a previously published method based on HPLC separation of OPA-derivatized basic amino acids was used with minor modifications (Teerlink et al. 2002; Andersson et al. 2010). All reagents and chemicals for the analysis of ADMA were from Sigma-Aldrich (Stockholm, Sweden). CRP and the daily blood sampling necessary for SOFA scoring (platelets, bilirubin, creatinine, and arterial blood gases) were analysed by standard procedures at the accredited University Hospital laboratory.

### Clinical outcome

Patient outcome three months after TBI was evaluated with the Glasgow Outcome Scale (GOS) (Teasdale and Jennett 1976). The GOS is a five-point scale where a score of 5 or 4 is defined as a favourable outcome and a score of 3, 2, or 1 as an unfavourable outcome.

### Statistics

The statistical software package Prism (version 5.0, GraphPad Software Inc., CA, USA) was used for statistical analyses. Mann-Whitney’s test was used for comparison of parameters between the groups (epoprostenol vs. placebo). The Kruskal-Wallis test, using Dunn’s multiple comparison test for post-hoc analysis, was used for comparison of parameters at different time points within each group. Fischer’s exact test was used for comparison of severe organ failure, antibiotic treatment, multi-trauma, and surgery between the groups (epoprostenol vs. placebo). Statistical significance was defined as *p* <0.05. Data are presented as mean ± SD, median (range), or per cent (%).

## Results

### Patient characteristics

In total, 89 patients with severe TBI with or without related traumatic injuries were admitted to Umeå University Hospital from 2002 to 2005. However, only 48 individuals fulfilled the inclusion criteria for the study. Exclusion was due to a GCS score >8 at the time of sedation and intubation (n = 18), too young or too old (n = 6), CPP <10 mmHg at insertion of the intracranial pressure monitoring device (n = 6), arrival >24 hours after TBI (n = 3), denial of inclusion (n = 2), or for other reasons, for example, no research staff available at the time of inclusion (n = 6). None of the patients were excluded due to discharge from ICU within 72 h. The study flow-chart is described in detail in the primary end-point study (Olivecrona et al. 2009). In two patients, no samples for cytokines were obtained so the results in this study are based on 46 patients randomized to 23 in the epoprostenol group and 23 in the placebo group.

No significant differences in baseline values were seen between the two groups regarding age, sex, GCS, ISS, AIS head, APACHE II, number of multiple injuries, number of patients receiving antibiotics, number of patients in need of surgery, or time to start of the study infusion (Table 1). In the placebo group, only neurosurgery (craniotomy) was performed. In the epoprostenol group, facial fracture reconstruction (three patients), and major orthopaedic surgery (two patients) were also performed in addition to neurosurgery (Table 1). In the epoprostenol group, 6 patients had one or two dilated pupils at the time of endotracheal intubation, i.e. at the time of first GCS score, compared to 5 patients in the placebo group. The prophylactic use of antibiotics was common in both groups due to fractures of the skull or face and suspected or verified cerebral spinal fluid leaks. No verified infections were noted during the 5-day study period. There were no adverse effects as hypotension or thrombocytopenia in this study associated with the intravenous infusion of epoprostenol during or after the time of administration.

**Table 1:** **Basic characteristics of the study population**

Characteristic	Epoprostenol	Placebo	
Age, median (range)	29 (15–63)	34 (16–63)	ns
Gender, F/M, n	10/13	7/16	ns
APACHE II, median (range)	20 (12–29)	21 (14–32)	ns
GCS, median (range)	5 (3–8)	6 (3–8)	ns
ISS, median (range)	29 (9–50)	29 (9–43)	ns
AIS Head, median (range)	5 (3–5)	4 (3–5)	ns
Multi-trauma, n (%)	17/23 (74)	14/23 (61)	ns
Antibiotic treatment, n (%)	22/23 (96)	21/23 (91)	ns
Surgery, n (%)	17/23 (74)	19/23 (79)	ns
Time from accident to start of study infusion in minutes (mean ± SD)	1131 ± 345	987 ± 310	ns

Gender F = Female, M = Male.

APACHE II = Acute Physiologic and Chronic Health Evaluation II.

GCS = Glasgow Coma Scale at the time of sedation and intubation.

ISS = Injury Severity Score.

AIS = Abbreviated Injury Scale.

ns = non-significant.

### Markers for systemic inflammation and endothelial dysfunction

**Figure 1:**
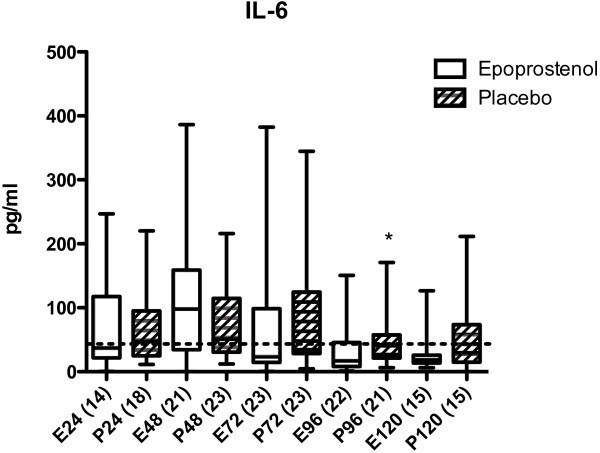
**IL-6 in pg/mL in patients treated with epoprostenol or placebo.** IL-6, IL-8, sICAM-1, CRP, and ADMA were measured ≤24 h, 25–48 h, 49–72 h, 73–96 h, and 97–120 h after trauma. These time ranges are denoted as 24, 48, 72, 96, and 120, respectively, in the figures. Values ≤24 hours were all sampled before the start of epoprostenol (E) or placebo (P) infusion. The numbers of patients at each time point are indicated in parentheses. Data are presented as Box plots. The horizontal line within the box indicates the median, the 25^th^ to 75^th^ percentiles are within the box, and the minimum and maximum values are shown with error bars. Dotted line = 43 pg/mL (reference value for IL-6 according to the manufacturer). *Statistically significant difference between the epoprostenol and the placebo group within 73–96 hours after trauma (p = 0.04, Mann-Whitney’s test).

**Figure 2:**
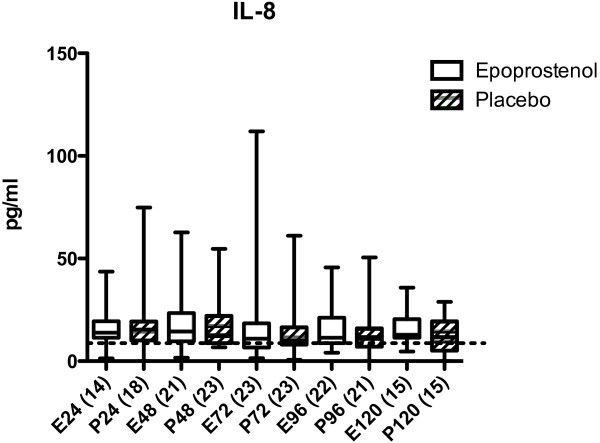
**IL-8 in pg/mL in patients treated with epoprostenol or placebo.** Dotted line = 8.6 pg/mL (reference value according to the manufacturer). For details see legend to Figure 1.

**Figure 3:**
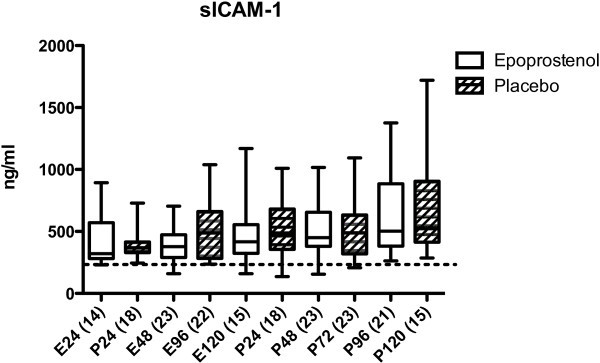
**sICAM-1 in ng/mL in patients treated with epoprostenol or placebo.** Dotted line = 230 ng/mL (reference value according to the manufacturer). For details see legend to Figure 1.

**Figure 4:**
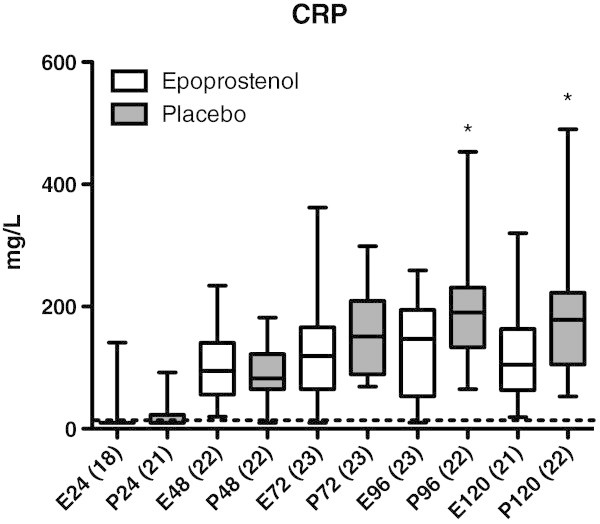
**CRP in mg/L in patients treated with epoprostenol or placebo.** Dotted line = 10 mg/L (the reference value at the accredited laboratory at Umeå University Hospital is <10 mg/L). *Statistically significant difference between the epoprostenol and the placebo group within 73–96 h (*p* = 0.04, Mann-Whitney’s test) and 97–120 h after trauma (*p* = 0.008, Mann-Whitney’s test). For details see legend to Figure 1.

**Figure 5:**
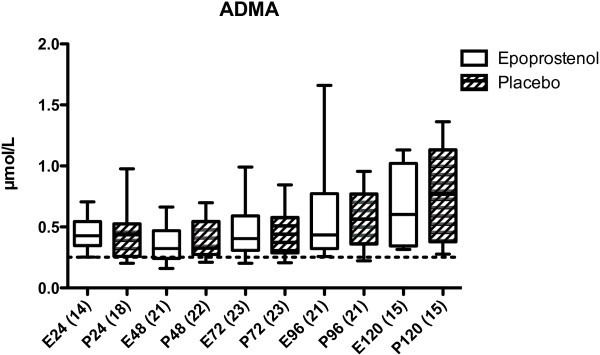
**ADMA in μmol/L in patients treated with epoprostenol or placebo.** No statistically significant differences were seen between the groups. ADMA levels in both groups were significantly higher 72 h, 96 h, and 120 h after trauma compared to the 24 h sample (*p* < 0.001, Kruskal-Wallis test). Dotted line = 0.25 ± 0.12 μmol/L (the mean ADMA level in a reference material of healthy subjects). For details see legend to Figure 1.

Before start of the epoprostenol/placebo infusion (first sample, ≤24 h after trauma), the levels of IL-6, IL-8, sICAM-1, CRP, and ADMA were not significantly different between the epoprostenol and the placebo group (Figures 1, 2, 3, 4 and 5).

In the epoprostenol group, IL-6 increased non-significantly from ≤24 h to 25–48 h after trauma. IL-6 was significantly lower 73–96 h after trauma in the epoprostenol group compared to the placebo group (Figure 1). There were no statistically significant differences in IL-8 or sICAM-1 concentrations within or between groups 0–120 h after TBI (Figures 2 and 3). CRP increased significantly from ≤24 h to 25–48 h after TBI in both groups (*p* <0.05, Figure 4). The epoprostenol group had significantly lower CRP levels at 73–96 h (*p* < 0.05) and 97–120 h (*p* < 0.01) after TBI compared to the placebo group. There was a significant increase in ADMA levels over time in both groups, but there was no significant difference between the epoprostenol and the placebo groups (Figure 5).

### Organ failure

There were no statistically significant differences in the frequency of organ failure between the groups according to SOFA score on days 1–5 (Table 2).

**Table 2:** **Severe organ failure, measured with the Sequential Organ Failure Assessment (SOFA) Day 1–5**

Organ system	Epoprostenol	Placebo	
Respiration	30/111 (27%)	32/113 (28%)	ns
Circulation	8/112 (7%)	10/114 (9%)	ns
Coagulation	6/114 (5%)	1/112 (1%)	ns
Liver function	0 (0%)	0 (0%)	ns
Renal function	0 (0%)	0 (0%)	ns

Number of days with SOFA ≥3 of all registered SOFA scores on days 1–5 in different organ systems are given in numbers and percentage.

The SOFA scores the degree of organ failure in six different systems (respiratory, haematological, hepatic, cardiovascular, renal, and central nervous system) from zero (no organ failure) to four (severe organ failure). The worst parameter per day in each system was registered without consideration for duration. Severe organ failure is defined here as SOFA ≥3. Central nervous system SOFA was not scored due to the fact that the patients were treated with continuous intravenous sedation.

ns = non-significant.

### Outcome

There was no significant statistical difference in outcome between the groups three months after TBI, and 13/23 (57%) of the patients in the epoprostenol group had a favourable outcome (GOS 4 or 5) compared to 11/23 (48%) in the placebo group. Mortality three months after TBI was 9% (2/23) in the epoprostenol group compared to 17% (4/23) in the placebo group.

## Discussion

This is the first clinical trial to investigate the systemic inflammatory modulating effect of the endogenous substance prostacyclin in a double-blinded randomised study in patients with trauma and severe TBI. The main endogenous role of prostacyclin is thought to be regulation of the interactions between the vascular endothelium and the blood to prevent aggregation and adhesion of platelets and leucocytes. Treatment with prostacyclin theoretically possesses the potential to influence the inflammatory response (Moncada et al. 1976; Jones and Hurley 1984; Scheeren and Radermacher 1997).

All patients in this study had a considerable inflammatory response, as has been previously described in patients with TBI (Stanisic et al. 2012). CRP levels increased significantly in both groups early after TBI. Thereafter, CRP decreased more rapidly in the epoprostenol group resulting in a significantly lower CRP level at 73–120 hours after TBI in the epoprostenol group compared to the placebo group.

The level of IL-6 correlates with the degree of inflammation, and serum levels of IL-6 have been shown to correlate with severity of injury, the occurrence of multi-organ failure, and to outcome (Jawa et al. 2011). Prostacyclin had a limited modulating effect on IL-6 in this study. IL-6 concentrations initially increased non-significantly in the epoprostenol group, but thereafter decreased rapidly. An early increase, followed by rapid decrease, in IL-6 levels after prostacyclin administration has been observed previously in an experimental study (Ohta et al. 2005).

In this study, IL-8 levels were not significantly different between the epoprostenol and the placebo groups. Because prostacyclin has a more pronounced endothelial effect, the influence on the chemotactic IL-8 cytokine might be small or non-existent (Charo and Ransohoff 2006).

The inflammatory marker sICAM-1, which mediates the translocation of leucocytes through the endothelium, did not increase after TBI nor was it affected by prostacyclin in this study. This could be due to the fact that sICAM-1, as a marker for endothelial dysfunction, is not substantially expressed after traumatic injuries (Du Clos 2000).

Elevated levels of ADMA, which inhibit the production of nitric oxide, lead to impaired endothelium-dependent vasodilation and increased leucocyte/platelet adhesion (Böger 2003; Siekmeier et al. 2008). No reports exist concerning the occurrence of endothelial dysfunction, as measured with ADMA, after TBI. In this study, ADMA increased significantly in both groups, and this indicates that a systemic pro-inflammatory response might induce endothelial dysfunction.

Severe trauma induces a systemic inflammatory response that can lead to capillary leakage and circulatory instability (Lenz et al. 2007; Stahel et al. 2007). Prostacyclin would theoretically be able to decrease capillary leakage, interstitial oedema, and the development of organ failure. In a previous experimental study, prostacyclin reduced volume loss after lipopolysaccharide-induced meningitis (Jungner et al. 2009). However, in the present study, even though prostacyclin had a modulating effect on CRP and IL-6 no difference was found in the incidence of organ failure.

Reduction of the inflammatory response after TBI has been tested using glucocorticoids as a pharmacological agent. Even though factors other than the anti-inflammatory effect of glucocorticoids could be responsible for the reduced inflammatory response, the CRASH study showed that corticosteroids were actually associated with a higher risk of death than placebo in TBI patients (Roberts et al. 2004). This implies that excessively inhibiting the systemic inflammatory response is not associated with a favourable outcome in TBI patients. Prostacyclin, with a half-life of a few minutes and with a distinct endothelial point of action, could be an alternative as modulator of the systemic inflammatory response, but further studies are needed.

This is a study with a limited number of patients. The dose of epoprostenol in this study could have been too low and/or the treatment time too short to have had a more prominent effect on the systemic inflammatory response. To what extent a decrease in the systemic pro-inflammatory response might be beneficial for patients with traumatic injuries, including severe TBI, remains to be investigated.

## Conclusions

In this randomised placebo-controlled clinical study, the prostacyclin analogue epoprostenol significantly modulated and reduced the systemic pro-inflammatory response as measured by CRP and, to a lesser extent, IL-6 levels in patients with severe TBI compared to placebo.
